# Early Diagnosis of Left Pulmonary Artery Sling During First Week of Life in a Term Baby Boy: A Case Report

**DOI:** 10.7759/cureus.6889

**Published:** 2020-02-05

**Authors:** Dima Kayal, Samer Minkara, Faissal Tleiss

**Affiliations:** 1 Pediatrics, Lebanese University, Beirut, LBN; 2 Internal Medicine, Beirut Arab University, Beirut, LBN; 3 Neonatology, Nini Hospital, Tripoli, LBN

**Keywords:** pulmonary artery sling, vascular anomaly, respiratory distress, atelectasis, surgical repair

## Abstract

Pulmonary artery sling is a rare cause of neonatal respiratory distress. Most patients with pulmonary artery sling present in early infancy with stridor and signs of respiratory distress. Diagnosis of pulmonary artery sling, like other vascular ring anomalies, can be made using various imaging modalities, and management encompasses urgent surgical repair as a definitive treatment. This is the first paper to report a successfully managed case of an early detected left pulmonary artery sling during the first week of life in a term male patient and to evaluate the diagnostic characteristics in alliance with it. CAse REports (CARE) guidelines were followed for reporting our case. In brief, a case of full-term baby boy was born by normal vaginal delivery and shortly after birth, the baby started to have respiratory distress not improving on O_2_. Chest X-ray revealed right upper lobe atelectasis which persisted despite mechanical ventilation and antibiotics. A thoracic CT scan showed developmental malformation of left main pulmonary artery, confirming the diagnosis of “left pulmonary artery sling.” The baby was immediately operated. One week later, chest X-ray showed gradual improvement and the baby was discharged home with no postoperative complications. Hence, we suggest that pulmonary artery sling should be suspected in any neonate with respiratory distress and unilateral lung field opacification. The fact that there are only very few reports on this disease raises a need to establish and implement well-defined guidelines and criteria for early diagnosis and management of pulmonary artery sling among newborns.

## Introduction

Pulmonary artery sling is a rare vascular anomaly where the left pulmonary artery arises from the right pulmonary artery (late bifurcation of the branch pulmonary arteries) [1]. The aberrant left pulmonary artery passes between the trachea and the esophagus prior to entering the left lung, leading to compression of the trachea and/or esophagus [2]. Associated with this are signs that include respiratory distress manifested as stridor, recurrent pneumonia, wheezing, and cyanosis, typically occurring within the first months of life. Indeed, when a sling compresses the trachea, it may produce obstructive emphysema, atelectasis of the right and left lungs, or both [3]. The prevalence of pulmonary artery sling is estimated to occur in one in every 17,000 school-aged children, while no data have been so far published concerning incidence and prevalence of pulmonary sling among infants [4].

The diagnosis of vascular ring can be made using various imaging modalities, including chest X-ray (CXR), CT scan, or MRI of the heart and major blood vessels, and echocardiogram [5], and management encompasses urgent surgical repair with a median sternotomy, cardiopulmonary bypass, and left pulmonary artery re-implantation [6].

Herein, a case of full-term baby boy was born by normal vaginal delivery and shortly after birth, the baby started to have respiratory distress not improving on O_2_. Routine blood tests were all normal. CXR revealed right upper lobe atelectasis which persisted despite mechanical ventilation and antibiotics. A thoracic CT scan was done and showed developmental malformation of left main pulmonary artery, confirming the diagnosis of “left pulmonary artery sling.” To the best of our knowledge, this is the first case to report diagnosis of pulmonary sling at this early age during first week of life. The case report was conducted and reported in accordance with CAse REports (CARE) guidelines for reporting case reports.

## Case presentation

This is a case of a 42-week-old gestational age baby boy, born via normal vaginal delivery to a 21-year-old healthy mother (G1P1A0) and a 25-year-old healthy father, with no history of consanguinity or a family history of vascular anomalies. Pregnancy was well followed with no complications, including no gestational diabetes or hypertension reported. TORCH screen results were negative in the first trimester and group B streptococcus (GBS) test was negative as well. There was no history of any drug intake during pregnancy except for folic acid and multivitamins.

The baby male was born with an APGAR score of 7 and 8 at 1 and 5 min, respectively. Shortly after birth, the baby started having respiratory distress reflected by subcostal retractions with desaturations not improving on O_2_ by nasal cannula, but no other signs were reported. So, he was transferred to the neonatal intensive care unit (NICU) where he was intubated, ventilated, sedated, and given one dose of beractant (Survanta; 4 mL/kg).

**Figure 1:**
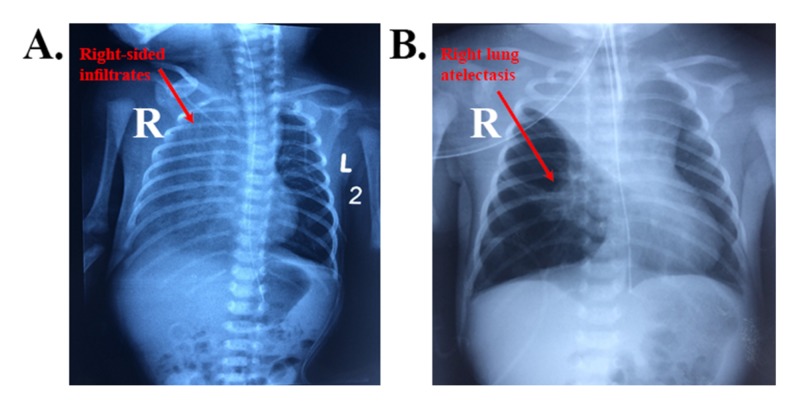
Chest X-rays revealing right-sided infiltrates (A) and right lung atelectasis (B). "R" signifies right side and "L" signifies left side

Physical examination revealed a hypotonic baby in moderate distress (nasal flaring and subcostal retractions) with no dysmorphic features, heart murmurs, or extremity edema. The remaining of the physical examination was normal. The differential diagnosis included stridor, tracheal stenosis, tracheomalacia, double aortic arch, pulmonary artery sling, and most importantly sepsis. Immediate full sepsis workup was started (except lumbar puncture as the baby was in unstable condition). Routine blood tests were unremarkable with white blood cell (WBC) count of 11,900 per microliter of blood (normal 4,000-10,000 per microliter of blood), neutrophils of 52% (normal 55%-70%), and negative C-reactive protein (CRP; normal <10 mg/L). Arterial blood gas (ABG) was performed also and turned to be normal upon admission except for low PaO_2_ [pH=7.35 (normal 7.38-7.42), PaCO_2_=38.5 mmHg (normal 38-42 mmHg), PaO_2_=52 mmHg (normal 80-100 mmHg), HCO_3_=21.5 mEq/L (normal 22-28 mEq/L), and SaO_2_=95% (normal 94%-100%)]. Other routine blood tests (complete blood count with differential and electrolytes) were all done and were normal. CXR revealed right-sided infiltrates with right lung atelectasis (Figure 1) and echocardiography showed mild infrasystemic pulmonary hypertension. Brain ultrasound was normal.

Broad spectrum intravenous (IV) antibiotic therapy, including ampicillin, gentamycin, and cefotaxime were started. Clinically, the baby was deteriorating with time. ABG was repeated showing increasing respiratory acidosis (pH=6.8, PaCO_2_=146 mmHg, PaO_2_=72 mmHg, HCO_3_=28.5 mEq/L, and SaO_2_=76%) despite strict mechanical ventilation. Laboratory workup (routine blood tests) was repeated and remained within normal range. Pressure of mechanical ventilation (SIMV mode) was increased causing slight improvement in the ABGs. CXR was done revealing persistence of the atelectasis in the right lung and the right-sided infiltrates. However, tracheal culture results revealed klebsiella pneumonia ESBL, so antibiotics were switched to imipenem with vancomycin, without any improvement.

**Figure 2:**
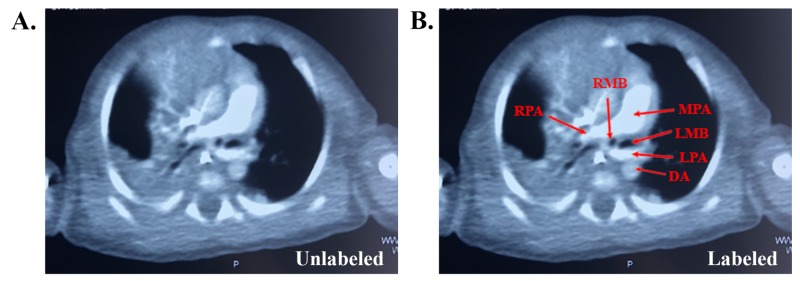
Thoracic CT scan showing developmental retro-tracheal malformation of the left main pulmonary artery originating from the right with compression of the origin of the right main bronchus (unlabeled in A and labeled in B). DA, descending aorta; LMB, left main bronchus; RMB, right main bronchus; RPA, right pulmonary artery; LPA, left pulmonary artery; MPA, main pulmonary artery

On day 9 of admission, thoracic CT scan was done showing developmental retro-tracheal malformation of the left main pulmonary artery originating from the right with compression of the origin of the right main bronchus, leading to right upper lobe collapse and right lower lobe infiltrations (Figure 2).

**Figure 3:**
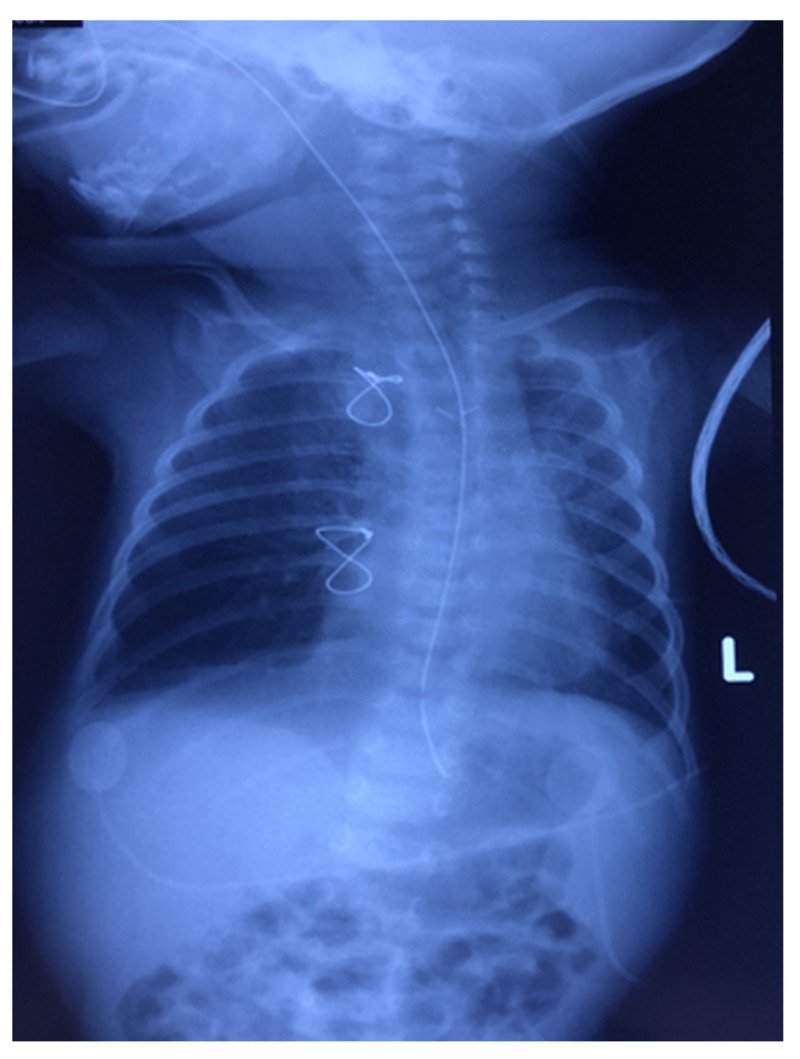
Chest X-ray showing gradual improvement of the patient with minimal right-sided infiltrates and absence of lung atelectasis. "L" signifies left side

The baby was diagnosed with “left pulmonary artery sling” and was immediately operated for this congenital vascular malformation. One week later, CXR showed gradual improvement (Figure 3) and ABGs were back to normal also. The baby was thus discharged home. Follow up after two months revealed full recovery with no complications reported.

## Discussion

The first report of pulmonary vascular slings was published in 1958 by Contro and colleagues as a rare cause of congenital bronchial obstruction [7]. However, anomalous left pulmonary artery was first described on autopsy by Glaevecke and Doehle in 1897 [8]. In normal physiologic formation of the human embryo, development of the great vessels commences at 20-22 days with the appearance of the six arches and it regresses in a craniocaudal fashion [9]. In brief, the left post-branchial vessels join the left sixth branchial arch giving rise to the left pulmonary artery whereas the right post-branchial vessels follow a similar sequence of events on the right sixth branchial arch to form the right pulmonary artery [10].

Left pulmonary artery sling occurs when the left post-brachial vessels fail to connect with the left sixth branchial arch [11], where the connection occurs with the right sixth branchial arch dorsal to the trachea forming what is referred to as a “pulmonary artery sling” [12]. As pulmonary artery sling is relatively rare, its incidence and frequency in the United States and globally have not been identified yet [13]. Moreover, reaching a definitive diagnosis is really challenging as clinical presentation and signs can be variable and nonspecific.

Most patients with pulmonary artery sling present in early infancy with stridor and signs of respiratory distress. Other clinical manifestations may include dysphagia (which is not much common) due to compression of the esophagus, failure to thrive, and recurrent chest infections [4]. In some reports, pulmonary artery sling remained asymptomatic until childhood [14]. Indeed, patients may just complain of mild symptoms/signs and present in late childhood or adulthood with nonspecific respiratory symptoms like chest pain, cough, orthopnea, and exertional symptoms [15-16].

In our case, the patient had signs of respiratory distress in the first hours after birth, and the diagnosis was made early in the first week of life. Our case highlights the importance of clinical suspicion of pulmonary artery sling and determines the proper diagnostic test warranted for early diagnosis. This is extremely important as delaying the diagnosis prompts significant morbidity and mortality. Henceforth, it is crucial to have a possibility of vascular anomaly high on the differential in cases of respiratory distress.

Yet, pediatric radiologists need to familiarize themselves with the anatomic variants that can result in a symptomatic vascular ring to avoid missing the diagnosis and eventually delaying immediate surgical repair [17]. Pulmonary artery sling can be diagnosed with prenatal ultrasound using the three-vessel trachea view and subsequent fetal echocardiography with particular attention to the relationship of the aortic arches, ductal arches, and the trachea [18]. Echocardiography is also a very helpful noninvasive screening technique that could be used as a first-choice modality for the diagnosis of pulmonary artery sling [19]. A more specific imaging technique that is substantial for final diagnosis and pre-operation evaluation is the contrast-enhanced CT which clearly shows the anatomy of pulmonary artery sling and the position and extent of trachea compression [19]. When a suspicion of vascular compression of the trachea is present, a barium swallow can be performed to visualize as anterior indentation of the esophagus on the lateral projection [20].

To manage pulmonary artery sling, surgical repair is the treatment of choice [4]. Two treatment approaches are present which depend on the presence and degree of tracheal stenosis and any other associated cardiac anomalies [4]. Surgery is usually performed either with a median sternotomy followed by cardiopulmonary bypass and left pulmonary artery re-implantation (which was the technique employed in our patient) [6] or thoracotomy without cardiopulmonary bypass [4], resulting in uniformly patent left pulmonary arteries. In general, the need for tracheal surgery stratifies patients into those with a good vs. bad prognosis, where reports show that patients with pulmonary artery sling who do not require tracheal surgery have favorable clinical outcomes. However, mortality can be reduced with the slide tracheoplasty technique and multidisciplinary team approach. Besides, survival beyond one-year post surgery signifies an excellent prognosis.

## Conclusions

In conclusion, death can occur in the early months of life among patients with pulmonary sling and survival is unlikely without surgical intervention. It is thus highly recommended that infants with recurrent respiratory signs and symptoms, such as chronic cough, stridor, wheezing, respiratory distress, and chest infections, be examined for the possible presence of congenital pulmonary artery sling. Besides, pulmonary artery sling should be suspected in any neonate with a unilateral lung field opacification. Early surgical management of symptomatic patients is the most effective way to treat congenital pulmonary artery sling.
